# Expanding the Social Care Network: A Review of Ohio’s Healthy Aging Initiative

**DOI:** 10.1093/geroni/igaf122.3430

**Published:** 2025-12-31

**Authors:** Bailee Brekke, Robert Applebaum, Jennifer Heston-Mullins

**Affiliations:** Miami University, Waddington, New York, United States; Miami University, Oxford, Ohio, United States; Miami University, Oxford, Ohio, United States

## Abstract

For older adults with a disability, Medicaid provides the majority of support for long-term services and supports. However, only 10% of individuals age 65 and older are financially eligible for Medicaid. Older Americans Act funding has failed to keep up with the growth in the older population and inflation, creating a service gap for older adults who are not eligible for Medicaid. As a result, states have explored other avenues of funding for social care services. Ohio recently allocated $40 million in county-level funding for older adults in need of social care in their community. Each county was required to spend 20% of authorized funds on housing and food insecurity and 10% towards internet access/digital literacy. The remaining 50% could be spent on services that met the unique needs of each county. Overall, the program served more than 130,000 individuals across Ohio. A review of program reporting showed that counties utilized their discretionary funds in such areas as health and wellness programs (n = 48 counties, mean expenditure=$29,250), transportation services (n = 41 counties, mean expenditure=$45,780) and strategies to address social isolation (n = 36 counties, mean expenditure=$93,690). Qualitative interviews with 27 county officials also provided insights into innovative service strategies and cross-sector collaborative efforts, such as partnerships with law enforcement and local businesses. Findings from this study highlight the importance of offering non-Medicaid funded services to support older adults to remain independent in their local communities.

